# The AGESW Pre-Dissertation Fellows Program: Participants’ Perceptions of Mentorship and Training for Academia

**DOI:** 10.1093/geroni/igaa057.1795

**Published:** 2020-12-16

**Authors:** Noelle Fields, Allison Gibson, Stephanie Wladkowski, Cara Wallace, Abigail Latimer

**Affiliations:** 1 The University of Texas at Arlington, Arlington, Texas, United States; 2 University of Kentucky, College of Social Work, Lexington, Kentucky, United States; 3 Eastern MIchigan University, Ypsilanti, Michigan, United States; 4 Saint Louis University, St. Louis, Missouri, United States; 5 University of Kentucky College of Social Work, Lexington, Kentucky, United States

## Abstract

Good mentoring is key for doctoral student success. In 2010, AGESW began offering the Pre-Dissertation Fellows Program (PDFP) to enhance social work doctoral students’ professional development and skillset for academia. The purpose of this study was to examine student participants’ perceptions of the PDFP in its role to providing mentorship and training for an academic position. This qualitative study examined eight cohorts (2010-2018) of the AGESW PDFP (N=85). Using thematic analysis, responses identified a number of aspects of professional development gained, gratitude for the training, an appreciation for candid advice received, and areas of professional development they felt they were lacking within their doctoral training. Findings bolster support for structured programs and professional development that supplement doctoral education in a student’s first two years. Implications for doctoral education, mentorship training, and avenues to enhance the AGESW pre-dissertation program will be discussed

